# Upcycling of Whole Pisco Grape Pomace: Influence of Emerging Extractions on Antioxidant Potential and Functional Quality of the Lipophilic Fractions

**DOI:** 10.3390/molecules30183776

**Published:** 2025-09-17

**Authors:** Issis Quispe-Fuentes, Fátima Rodríguez-Ramos, Jacqueline Poblete, Iván Leyton-Valle, Elsa Uribe

**Affiliations:** 1 Food Engineering Department, Universidad de La Serena, Av. Raul Bitran 1305, La Serena 1700000, Chile; fatimajrb@gmail.com (F.R.-R.); j.pobletegalleguillos@gmail.com (J.P.); ivaan.vleyton@gmail.com (I.L.-V.); muribe@userena.cl (E.U.); 2 Faculty of Medicine and Health Science, Universidad Central de Chile, La Serena 4484525, Chile

**Keywords:** antioxidants, fatty acids, lipophilic fractions, non-conventional extraction, pressurize extraction, randall

## Abstract

This work aimed to promote an integrated valorization approach for recovering lipophilic fractions from whole pisco grape pomace using emerging extraction technologies such as Randall (40 °C, 360 min) and Pressurized Liquid Extraction (PLE: 60 °C, 10 min) to characterize lipid-soluble biocompounds and evaluate their functional quality and antioxidant potential. PLE achieved maximum extraction yields (11–15%). Extraction techniques did not significantly alter fatty acid profiles, with C18:2n6c linoleic acid being most abundant (65–69%), followed by C18:1n9c oleic acid (17–21%), while C20:1 eicosanoid acid was least prevalent (1–2%). The fatty acid profile enabled functional potential evaluation through atherogenicity, thrombogenicity, and hypocholesterolemic/ hypercholesterolemic ratios, showing values comparable to other lipid sources. α-Tocopherol content was significantly influenced by extraction techniques (87–645 μg/g), while polyphenol content showed no significant differences between methods (0.6–0.8 mg GAE/g extract). Randall demonstrated higher DPPH radical scavenging capacity (25–30%), while PLE presented higher ORAC values (68–120 μmolTE/g extract). This represents the first characterization of lipophilic fractions from this pomace type, highlighting how emerging extraction techniques affect recovery of high-quality, antioxidant-rich fractions. Results suggest their potential as functional biocompound sources and candidates for various applications, contributing to grape waste valorization strategies.

## 1. Introduction

Chile is the seventh country among the leading wine-producing countries in the world [1], but pisco production is also relevant after wine manufacturing, with pisco being a distillate alcoholic beverage protected by a designation of origin, produced in northern Chile by specific grape varieties. Its manufacturing has implied the generation of large quantities of by-products, such as pomaces and stems, among others. According to Martinez et al. [2], the Chilean pisco and wine industry generates about 400,000 tons/year of grape pomace as organic waste, one of the most abundant solid by-products during these processes for pisco and wine [3], where 30% *w*/*w* as organic material has been associated with the pisco production, and nearly 6000 tons/year of pomace are estimated to be eliminated annually just from this industry [4]. Some scientific achievements in the reuse of pisco grape pomace have been focused on its use as fertilizer for the generation of compost at a small scale [2]; its chemical characterization to establish the nutritional composition and antioxidant properties [2,4,5]; or even the use of drying technologies to improve the retention of its bioactive compounds [4], while the use of other by-products of this industry, such as vinasse, have been used for scientific developments to obtain biofilms [6], and the treatment of this by-product by ultrasound and heterogeneous photocatalysis [7,8,9]. Nevertheless, other valorization strategies are urgent and still necessary to decrease the volume of the waste generated, considering the great potential of the chemical composition of these matrices. Furthermore, in line with global strategies to reduce waste generation highlighted by the United Nations in the 2030 Agenda for Sustainable Development, circular economy strategies such as prevention, reduction, recycling, and reuse are recommended [10].

Studies about extraction/identification strategies of lipophilic fractions from pisco grape pomace that allow the establishment of the functional properties and antioxidant profile, considering non-polar bioactive compounds and promoting their recovery, are scarce today. It is well established that, in general, the grape pomace has a wide profile of bioactive compounds improving its antioxidant potential [8,11,12]. However, there are also several relevant non-polar components; among them, essential unsaturated fatty acids, primarily linoleic and linolenic acids, besides carotenes or tocopherols, have been extensively reported in grape seed oils [13,14]. A wide list of studies has shown the relevant role of essential fatty acids in the correct performance of metabolism, highlighting their hypolipidemic, anti-inflammatory, antithrombotic, antiatherogenic, and antiarrhythmic properties [7,8,9]. On the other hand, grape seed oil is also an excellent source of vitamin E and high amounts of tocopherols and tocotrienols [15].

In the valorization of grape pomaces, lipophilic fractions are usually obtained mainly from grape seeds [15,16,17]. But valorizing the whole grape pomace to obtain lipophilic fractions can offer several advantages as synergistic effects by the combination of different compounds from grape skin, seeds, and stems. This enhances the bioactive potential, maximizing the waste reduction and sustainability thanks to the valorization of the entire matrix or by diversifying the nutrient profile, taking advantage of a wider range of bioactive compounds compared to exclusively grape seeds. The quality and functional properties of the lipophilic fractions obtained from whole grape pomace will depend on the grape variety or the agroclimatic growing conditions. However, the extraction method and conditions are imperative in this process. Considering this, there are some techniques called “novel and non-conventional techniques” that have emerged as suitable alternatives to overcome the limitations of conventional techniques, considering physical ones, such as cold-pressing, and chemical ones, such as Soxhlet, which are often highly costly, and display longer extraction times, or low extraction yields [18,19]. Additionally, this growing interest has also been based on the development of safer and more efficient techniques that balance economic, social, and environmental factors in line with green chemistry principles, aiming to reduce or eliminate the use of harmful solvents and products that pollute the environment [20]. These include pressurized fluids, deep eutectic solvents, bio-based solvents, and auxiliaries such as microwave, pulse electric field, and ultrasound [21].

To overcome these issues, alternative non-conventional extraction methods have been evaluated, which are faster, more selective, efficient, and generally clean solvents. Among these methods are Pressurized Liquid (PLE), Supercritical Fluid (SFC-CO_2_), or ultrasound-assisted extraction [22,23], where PLE has been considered a novel extraction technique that uses common solvents at high pressures and temperatures (typically between 50 and 200 °C and 10–15 MPa) [24], reaching rapid and effective extractions using a reduced amount of solvent volume [25,26]. In general, ultrasound-assisted and pressurized solvent extractions are the most frequently studied for these type of matrices, highlighting that emerging methods are not necessarily more efficient than the classical ones but are generally less time- and organic-solvent-consuming [27].

More studies about lipophilic fractions from pisco grape pomaces need to be conducted where their functional and antioxidant potential can be shown, thus constituting this fact as a novelty of the research. For this reason, the main objective of this work was to promote an integrated valorization approach with the recovery of lipophilic fractions from whole pisco grape pomaces, comparing extraction technologies, to characterize the main lipid-soluble biocompounds as fatty acids and α-tocopherol, and their associations with relevant quality and functional indicators as atherogenicity (AI) and thrombogenicity (TI) index, establishing the functional and antioxidant profile. This work seeks to encourage the total reuse of this by-product, giving added value and providing information for sustainable exploitation, as a valuable approach to reduce the environmental impact, and a way to promote a circular economy in the pisco industry.

## 2. Results and Discussion

### 2.1. Influence of Different Extraction Techniques on Total Extraction Yield

Table 1 depicts the results of the extraction yield for each technique studied in the obtention of pisco lipophilic fractions. Regardless of the grape variety mixtures, the yields obtained were not similar among harvests. This difference could probably be attributed to the different seed/pomace proportions and unequal lipid content of each mixture. Comparing yields between different extraction techniques, for harvest 2023, no statistically significant differences between techniques were observed, in comparison to harvests 2019 and 2021. And in general terms, when yields were compared, Randall showed lower extraction yields than PLE. These results could be correlated with the fundamental differences between extraction methodologies. Randall extraction, being a modified version of Soxhlet, employs prolonged extraction times and high temperatures that typically increase the likelihood of thermal degradation of biocompounds, resulting in lower extraction yields [28,29]. In contrast, PLE operates under a combination of high pressures and temperatures that proves beneficial for biocompound preservation and consequently enhances yield. The synergistic effect of high temperature and high pressure applied for short periods facilitates both solubility and mass transfer rate from the matrix to the solvent, leading to increased solvent diffusivity and improved matrix kinetics [30,31]. Furthermore, high pressure ensures that the solvent remains in liquid form even at temperatures above its boiling point and below its critical point, which is decisive for maintaining efficient extraction conditions and optimizing yield [32]. Results for yields among grape pomace samples for PLE extraction showed statistically significant differences considering the mix of grape varieties present in the different harvests studied. Freitas et al. [33] showed for PLE (103 bar/25–100 °C/hexane) a similar yield (~ 9.4%) for seed oil from a mix of grape pomace containing Hebermont and Isabel varieties, considering that in our case the sample was the whole grape pomace and not only seeds usually rich in lipids. In general, the extraction yield of lipophilic fractions from plant materials can be influenced by several factors encompassing the properties of the plant material, the extraction method, and operational parameters.

### 2.2. Indicators of Quality for Pisco Grape Pomace Lipophilic Fractions

Parameters such as refractive index (RI), peroxide value (PV), and water activity (a_w_) were studied in pisco grape pomace lipophilic fractions and compared for harvest and extraction methods (Table 2). The RI is well-defined as the relationship between the rate of light in a vacuum and the rate of light in oil at a determined temperature [34]. RI also measures the purity and quality of oils, which is related to the degree of unsaturation [35,36]. Results for RI in pisco lipophilic fractions did not exhibit statistically significant differences between extraction techniques, with values close to the ranges for virgin and refined olive oil (1.4677–1.4705), or olive pomace oil (1.4680–1.4707) [31]. While a comparison with RI values for grape seed oil was made according to Vieira et al. [37] (Soxhlet: 1.4520–1.4760; pressing: 1.4640–1.4760), finding small differences, considering that our lipophilic fractions were obtained from whole pisco grape pomace and not only from the seeds.

The peroxide value (PV) is used as an indicator of the level to which rancidity reactions have occurred during storage, and it can be used as a signal of the quality and stability of fats and oils [38]. In general, PVs for pisco lipophilic fractions showed differences between extraction techniques, considering the effect of variables as time, temperature, and oxygen exposition on PV, when these techniques are used. Results for Randall were expected due to the high temperatures and long times compared with PLE, which can promote effects on lipid biocompound stability and stimulate oxidation. The reduction in the PV was marked by the harvests, decreasing by approximately 16–33% when PLE was applied. Water activity (a_w_) is a relevant parameter for fats and oils, affecting their stability. Also, a_w_ is considered a significant factor in preventing or limiting microbial growth [39]. The a_w_ values for lipophilic fractions ranged from 0.3741 to 0.4357, without significant differences between techniques. These values were analyzed based on the relationship between a_w_ and oxidation rate according to Vu et al. [35], considering that a_w_ is one of the factors that could have an impact on lipid oxidation in low-moisture foods, particularly in oils and fats. Lipid oxidation can be influenced by water activity because it affects the physical state of the product and the availability of reactants, considering that a_w_ 0.2–0.3 is associated with the lowest oxidation rates, while a_w_ 0.3–0.9 can lead to an increase in oxidation rates. The results of this study showed water activity values ranging from 0.3741 to 0.4357, within the range that promotes lipid oxidation and explains the peroxide values obtained. At these elevated water activity levels, oxidation rates increase due to the plasticizing effect of water on the food matrix, which enhances the mobility and interaction of oxygen, reactants, and catalysts, thus facilitating oxidation reactions [40].

### 2.3. Fatty Acid Profile and Functional Quality Indicators

**Figure 1:**
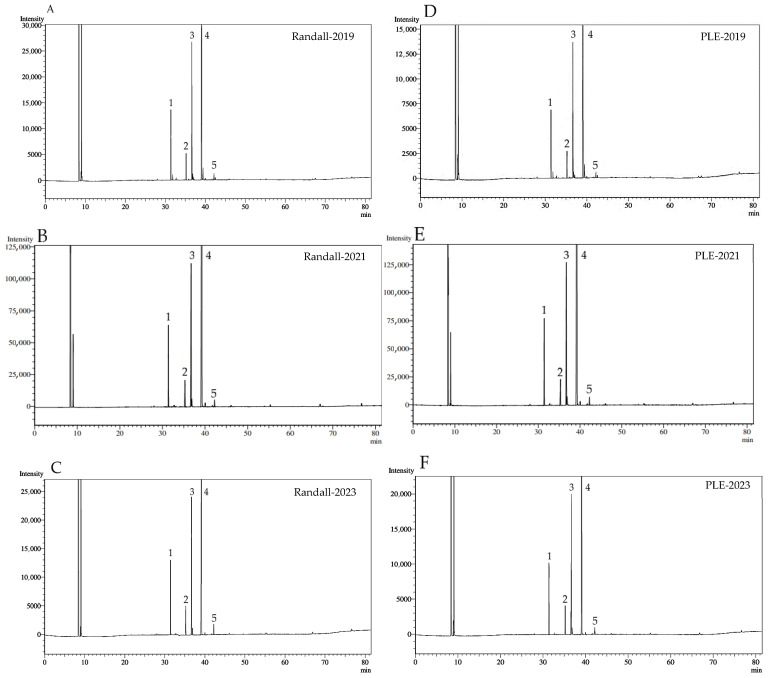
Chromatograms of FAMEs (Fatty Acid Methyl Esters). Lipophilic extract of pisco grape pomace from different harvests and extraction techniques. The graph represents one of the triplicate samples. PLE: Pressurized liquid extraction. (**A**) Randall- harvest 2019; (**B**) Randall- harvest 2021; (**C**) Randall- harvest 2023; (**D**) PLE- harvest 2019; (**E**) PLE- harvest 2021; (**F**) PLE- harvest 2023. Compounds: **1**: C16:0; **2**: C18:0; **3**: C18:1n9c; **4**: C18:2n6c; **5**: C20:1.

The fatty acid composition of the lipophilic fractions is shown in Figure 1 and Table 3. The lipophilic fractions contained saturated fatty acids (11.02–12.45%), monounsaturated fatty acids (19.43–21.96%), and polyunsaturated fatty acids (65.63–69.36%). Globally, the fractions depicted a small ratio S/U (Saturated FA/Unsaturated FA) between 0.12 and 0.14, without statistically significant differences between techniques. Linoleic acid (C18:2n6c) was the most abundant polyunsaturated fatty acid in the fractions, while eicosenoic acid (C20:1) was found in the lowest proportion. These results were comparable with data from Baydar et al. [36] for oils from both grape seed and pomace by Soxhlet extraction, obtaining for oil pomace levels of linoleic acids equal to 61.16–69.97%, with a clear dependence of grape variety, predominating white grapes, similar to our pomace mixture. Also, the results were compared with data from Dimić et al. [18] for antioxidant lipophilic fractions from only red grape seed mixtures, reporting a similar range for polyunsaturated fatty acids (69.27–74.23%).

Five fatty acids were detected in all samples, irrespective of the extraction method or harvest, considering fatty acids detected at concentrations above 0.5%, while those present in proportions below this threshold were not considered relevant for the purposes of the study. The fatty acid proportions showed statistically significant differences between Randall and PLE fractions for palmitic and stearic acid, but not for oleic, linoleic, and eicosenoic acids. Values with lowercase letters in Table 3 compare all extraction techniques and harvest for each fatty acid, showing statistically significant differences in some cases. Several indicators were established from the fatty acid composition of lipophilic fractions, and they are depicted in Table 4.

The atherogenicity index (AI) and thrombogenicity index (TI) can be useful indicators of several effects that fatty acids could have on human health and the probability of an elevated incidence of atherosclerosis, development of blood clots, atheroma, and thrombus formation [37]. Oils with lower values for AI or TI indicate that using them in the diet reduces the risk of cardiovascular diseases [41]. AI and TI can be estimated from the composition of fatty acids. AI and TI values were found in pisco lipophilic fractions in the ranges of 0.088–0.099 and 0.248–0.285, respectively. AI values were compared with data from Carmona-Jimenéz et al. [7] for grape pomace oils with AI 0.112–0.149 and grape seed oils (0.097–0.112). Also, Dimíc et al. [18] showed AI values only for red and white grape seed oils (0.081–0.090). No major differences were found between these studies and our results regarding the use of whole grape pomace or only grape seeds; this may be attributed to the fact that the main lipid contribution comes from the seeds, representing the fraction richest in oil from the pomaces. Alternatively, TI values were found to be near to data from Dimíc et al. [18] and Carmona-Jimenéz et al. [7] for grape seed oils, with 0.249–0.268 and 0.28–0.31, respectively. Additionally, relevant associations were made with the profiles of other known commercial oils. Our AI results were near chia seed (0.08), walnut (0.09), mustard (0.08), or pecan oil (0.07), while in terms of TI could be compared with almond (0.24), peanut (0.35), and avocado oil (0.39) [42].

The hypocholesterolaemic/hypercholesterolaemic (H/H) is a relevant marker used to evaluate their effect on cholesterol levels in the body, and it is especially significant when evaluating the health benefits of vegetable oils [43]. The H/H values for lipophilic fractions in harvests ranged from 10.018 to 11.931 (Table 4), obtaining near values when this ratio was compared between extraction methods. This could be attributed to the similarity among fatty acid profiles for lipophilic fractions. According to Dimíc et al. [18], high levels of this ratio are desirable for nutrition since they indicate the effect of fatty acids on cholesterol metabolism. Those authors showed near values oscillated from 11.07 to 12.28 for red grape, and 11.30 to 12.09 for white grape seed oils, suggesting that the fatty acid profile of the seeds used can be comparable, regardless of the variety or processing method. On the other hand, COX, or oxidability index, was also evaluated. This parameter indicates the tendency of nutritional oils to oxidize, and it is an important measure to evaluate the stability of oils [44], especially in cooking and storage. Oxidative changes are relevant since they induce deterioration of the nutritional values of oils, accompanied by secondary product formation, such as aldehydes and ketones, besides free radicals, which can lead to oxidative stress and organ disorders [38]. COX values ranged from 6.760 to 7.139, close to reported values for white and red grape pomace oil (6.87–7.24) and for tomato (8.11) and dill (7.25) seed oils [7,18,45]. The study of nutritional indicators of oil quality is generally relevant; it can provide guidelines to consider their application areas, especially those obtained from agro-industrial waste in the search for strategies to reduce the volumes generated from these discards, taking into account the valuable chemical composition of these matrices, which are often ignored.

### 2.4. α-Tocopherol Content

Tocopherols are fat-soluble compounds with effective antioxidant properties and a wide application as food ingredients, improving their oxidation stability [46]. Specifically, vitamin E is included in a group of tocochromanols [40], being α and γ-Tocopherol, the main forms of vitamin E in the diet [47]. According to Garavaglia et al. [13], grape seed oil has a great content of vitamin E, ranging from 1 to 53 mg/100 g of oil (10–530 μg/g), which is higher than other known matrices such as highly-consumed soybean and olive oil. Levels of α-tocopherol were studied in lipophilic fractions of pisco grape pomace, and the results are depicted in Table 5.

Statistically significant differences were depicted between extraction methods for α-tocopherol content in extracts. Also, differences were shown among harvests, where several additional factors could be influenced, such as agroclimatic conditions or endogenous levels. On the other hand, by comparing results between methods, the α-tocopherol content was significantly affected by the extraction techniques, for example, when the PLE method was used the samples depicted a decrease in α-tocopherol content equal to 53.7, 75.8, and 47.7% for harvest 2019, 2021, and 2023, respectively, compared with the Randall method, which showed better extraction yields. The degradation of thermolabile biocompounds, such as α-tocopherol, cannot be ruled out in the extraction methods used, highlighting that, in addition to the exposure to certain temperatures, a variety of other factors, such as UV light, can also degrade α-tocopherols [48]. In the case of the Randall scheme, the recirculation of the solvent and continuous heating can contribute to higher yield and broader contact with the molecules in the solid matrix. While in the PLE method, the elevated pressures used may be beneficial in the extraction of some analytes but may not always be efficient for delicate compounds like vitamin E [49], where its stability under pressure may be a factor to consider, especially in other techniques such as High Pressure Processing (HPP), affecting the structural stability of molecules depending on pressure range or molecule type [50]. But in the case of PLE, the stability of the molecules may be reduced due to the combination of high temperature and high-pressure conditions that can significantly decrease the strong interaction between the solute and the matrix by van der Waals forces, hydrogen bonds, or by dipole attraction between solute molecules and the actives sites of the sample matrix, improving the efficiency of extraction and lowering the viscosity of the solvent [51]. Dimić et al. [18] studied grape pomace oils and the impact of pressure on the yield extraction of tocopherols, concluding that the higher pressure reduced tocopherol content, but it gave higher oil extraction yields. It is important to note that these authors used Supercritical Fluid extraction. Although SFE requires higher pressures than those used for PLE, that data was helpful in our case for comparing the pressure effect on yield extraction.

### 2.5. Total Polyphenols Content (TPC)

The total polyphenol content present in the lipophilic fractions was evaluated and compared between extraction methods (Table 6). Despite the difference in polarity, certain polyphenols may be present in oils due to extraction processes that incorporate these compounds from the original plant. In addition, in our case, hexane-extractable polyphenols could be recovered in the pisco lipophilic fractions.

Generally, there were no statistically significant differences between the extraction methods for the recovery of polyphenols in the matrices. Results were compared with Bail et al. [37], based on the characterization of total polyphenols in grape seed oils by cold pressing process, ranging from 0.059 to 0.115 mg/g for nine grape varieties, finding the lower TPC values for refined and treated grape oils. These results showed that TPC values in vegetable oils are usually low, and the lipophilic fractions of pisco pomace were no exception. Although polyphenols are known antioxidant molecules, their antioxidant potential in this type of matrix can also be improved and complemented with bioactive compounds such as vitamin E, which are relevant in these matrices due to their chemical affinity.

### 2.6. Antioxidant Potential

**Figure 2:**
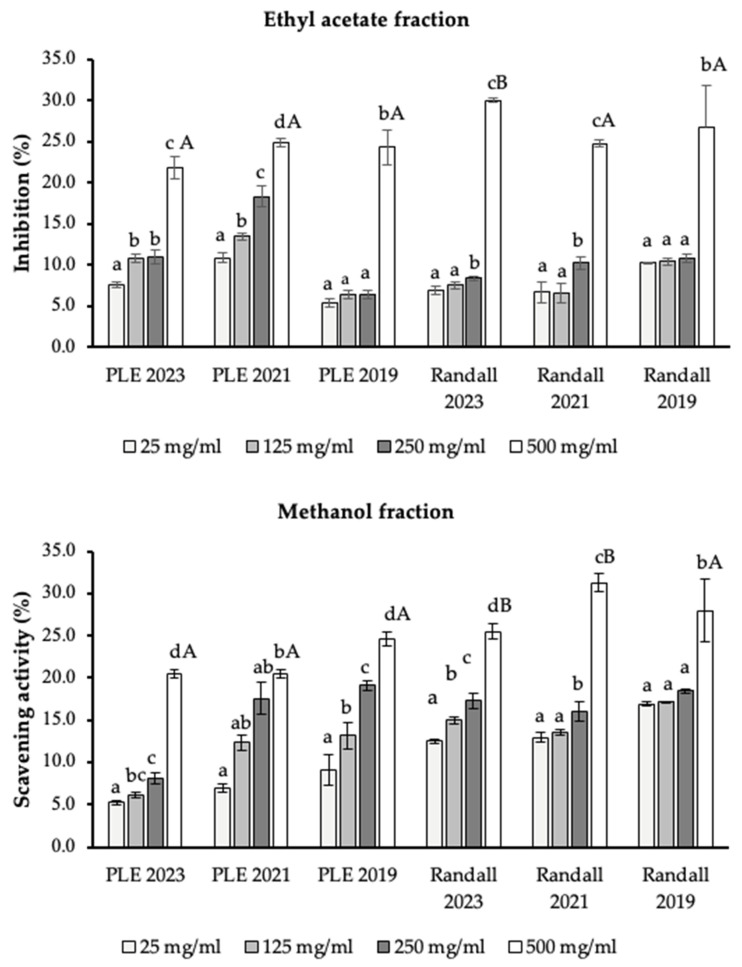
Antioxidant potential by DPPH assays of lipophilic fractions by Randall and PLE methods for different harvests. Values are expressed as means ± SD of triplicate. Different lowercase letters indicate comparison considering different concentrations of lipophilic fractions, and different uppercase letters the comparison for samples of 500 mg/mL for each extraction technique and harvest (*ρ* < 0.05). PLE: Pressurized Liquid Extraction.

The antioxidant potential of lipophilic fractions was assessed by DPPH and ORAC assays. The results for the DPPH assay are depicted in Figure 2.

Methanolic and ethyl acetate fractions were obtained using 5–100 mg of each extract to assess the inhibition rate as scavenging activity (%). The use of these two solvents with different polarities was intended to extract a broader range of antioxidant compounds from the oil matrix. This strategy allows the evaluation of antioxidant activity by capturing polar and less polar compounds. In this context, using the hexane extract as a base before fractionation with methanol or ethyl acetate is relevant as it helps isolate oil-soluble compounds, which are then separated into more specific polarity-based fractions [52]. The higher inhibition was obtained using 500 mg/mL of lipophilic fraction in both methanolic and ethyl acetate fractions, with statistically significant differences between 25, 125 and 250 mg/mL. Randall’s method showed a better performance for scavenging activity by DPPH radical, and it could be attributed to the fact that Randall showed better performance for the content of antioxidant molecules such as α-tocopherol. In addition, Randall proved its efficiency for recovering lipophilic fractions with a scavenging activity from 54% to 56% for the higher concentration used, considering the methanolic and ethyl acetate fractions, with IC_50_ values equal to 470–490 mg/mL. Fernandes et al. [53] showed values near those obtained from pisco grape pomace lipophilic extracts, depicting results for the DPPH scavenging effect between 38 and 59 % for grape seed oil from ten red grape varieties, finding relevant correlations between DPPH scavenging and the tocopherol content in samples.

The ORAC assay was also considered to complement the study of the antioxidant potential for pisco lipophilic fractions, where acetone, ethyl acetate, and *n*-hexane were used to fractionate the pisco fraction by polarity and to evaluate the distribution of antioxidant compounds (Table 7).

The polarity screening using different solvents showed that the *n*-hexane fraction depicted the highest value in most cases, with statistically significant differences in results for acetone and ethyl acetate. That behavior was expected considering that the antioxidant biocompounds present in pisco lipophilic fractions are non-polar (for example, vitamin E). That is relevant since especially fat-soluble antioxidants are essential in preventing the peroxidation of polyunsaturated fatty acids (PUFA) in biological membranes [46,54]. Comparing results between the extraction methodologies, PLE exhibited the highest ORAC values, being two and three times higher than the results achieved by the Randall method. This variance may be associated with the temperature and pressure conditions of PLE, which could increase the release of bound phenolic compounds from the pisco grape pomace matrix and reduce extraction time, consequently preventing oxidative degradation and contributing to improved oxidative stability. Results for *n*-hexane from pisco grape pomace lipophilic fraction were from 68.56 to 119.55 µmol TE/g of oil, a range higher than values reported by Prior et al. [54] with 24–67 µmol TE/g of oil for grape seed oil, extracted by different methods and conditions using Soxhlet, ultrasound, and press. The ORAC assay has the advantage that it can be performed for both the hydrophilic and lipophilic matrices. Therefore, the two values can be added together to obtain a total antioxidant capacity for the sample [55]. In our case, it can refer to the different solvents used, highlighting that emergent techniques such as PLE can preserve the antioxidant potential of fractions rich in non-polar biocompounds derived from grape pomace.

## 3. Materials and Methods

### 3.1. Plant Material

Three pisco grape pomaces provided by The “Compañía Pisquera de Chile” (Province of Limarí, Coquimbo Region, Chile) were studied from different harvest seasons with the following composition: Harvest 2019 Moscatel Austria (90%) and Pedro Jimenez/Rosada Pastilla (10%); Harvest 2021 Moscatel Rosada (60%) and Alejandria (40%); and Harvest 2023 Pedro Jiménez (77.4%), Moscatel Rosada (6.2%), and Moscatel de Alejandria (16.4%), all composed of seeds, skins, and, in minor quantities, stems. The grape pomaces were dried under optimized conditions according to previous publications from our research group, using vacuum drying at 60 °C for 6.5 h [4]. Then, the dehydrated grape pomaces were grinded in an analytical mill (IKA^®^ A-11, Wilmington, DE, USA), sieved using a 50 μm No. 35 sieve (ASTM E-11) to obtain homogeneous pisco grape pomace flours (PGPF), which were vacuum packed and stored in a dark and moisture-free place.

### 3.2. Extraction Techniques

#### 3.2.1. Randall Extraction Method

A Soxhlet extraction unit (Büchi E-812, Flawil, Switzerland) was used to obtain pisco lipophilic fractions using the Randall method (AOAC Official Method 920.172). The extraction assay was performed using 5.0 g of PGPF with 100 mL of *n*-hexane p.a. (MERCK, Darmstadt, Germany). The pisco lipophilic fractions were obtained using an immersion time of 30 min, a stirring time of 10 min, a washing time of 120 min, a recovery time of 20 min, and a cooling time of 20 min. Solvent was evaporated under vacuum at 40 °C/360 mbar. After that, the lipophilic fractions were stored in Eppendorf tubes at 4 °C until analysis.

#### 3.2.2. Pressurized Liquid Extraction (PLE) Method

The PLE scheme was carried out according to the methodology of Jin et al. [23]. For this, 10.0 g of PGPF was weighed, mixed with 10.0 g of diatomaceous earth (Celite 566, ENVIRO CLEAN^®^, UCT, Bristol, PA, USA), and placed in a 100 mL stainless steel cell with a glass filter. The cell was positioned in the Pressurized Liquid Extraction equipment (Dionex Accelerated Solvent Extractor 150, Thermo Scientific, Sunnyvale, CA, USA). 103 bar and 60 °C were applied, considering a performance of three cycles with *n*-hexane and an extraction time of 10 min each cycle. The rinse volume was 40%, and the purge time was 120 s. The extraction procedure was performed in triplicate. The extraction solvent was evaporated under a pressure of 360 mbar and at 40 °C. Once obtained, the lipophilic fractions were placed in Eppendorf tubes and kept at 4 °C until analysis.

Extraction experiments were performed in triplicate, and the results are expressed as the mean ± standard deviation of three independent extractions.

#### 3.2.3. Extraction Yield

The extraction yield for all lipophilic extractions was estimated according to the following Equation (1):

(1) $$EY\;\left(\%\right)=\frac{mass\;of\;the\;extracted\;oil}{\;mass\;of\;the\;grape\;pomace}\times 100$$

All determinations were performed in triplicate, and the results were expressed as mean ± standard deviation.

### 3.3. Chemical and Antioxidant Characterization of Lipophilic Fractions from Pisco Grape Pomaces

#### 3.3.1. Indicators of Quality

Refractive index (RI), peroxide value (PV), and water activity (a_w_) were measured in pisco lipophilic fractions. RI was determined according to AOAC 921.08 methodology. Refractive index [56], using an Abbe Refractometer with a digital thermometer (ATAGO, NAR-1T, Tokyo, Japan) with measurements at 50 °C, 55° C, and 60 °C. The PV was estimated by the methodology of AOAC 965.33 for the peroxide value of oils and fats [48], considering 0.2 g of sample and potassium iodide 0.01 N for titration. Finally, the a_w_ was measured at 25 °C using a water activity meter (AQUA LAB, model 4TE (Pullman, WA, USA), with 1 g of sample. All measurements were performed in triplicate, and values were expressed as mean ± standard deviation.

#### 3.3.2. Fatty Acid Profile

Using the methodology of Quispe-Fuentes et al. [57], the fatty acid profile of the lipophilic fractions of pisco was determined by sample derivatization through a transesterification reaction (saponification with 2 N KOH in methanol and *n*-hexane) to obtain fatty acid methyl esters (FAME). For this, 50 μL of lipophilic fractions were mixed with 200 μL of KOH/methanol (2 N), vortexing for 1 min, and subsequently extracting the fatty acid methyl esters with 500 μL of *n*-hexane (3 min stirring, 1 h standing). For the identification of FAMEs, gas chromatography was used (Shimadzu, model GC-2010, Shimadzu Corporation, Kyoto, Japan) with an autosampler, flame ionization detector (FID) at 250 °C, and external standards of commercial reference compounds (Sigma Aldrich, Milan, Italy). Chromatographic conditions included: initial temperature of 100 °C (13 min), ramping to 180 °C at 10 °C/min (held for 6 min), then to 200 °C at 1 °C/min (held for 20 min), and finally to 230 °C at 4 °C/min (held for 7 min). FAME samples (1 μL) were injected in split mode. The separation was performed using a BPX-90 capillary column (100 m × 0.25 mm inner diameter, 0.25 μm film thickness; SGE Analytical Science, Melbourne, Australia). All measurements were performed in triplicate, and the profile of results was expressed in percentage as mean ± standard deviation.

#### 3.3.3. Indicators of the Functional Quality

The atherogenicity index (*AI*) and thrombogenicity index (*TI*) were calculated based on fatty acid profile information, considering the importance of these parameters to measure the risk of contracting cardiovascular diseases [50,51]. *AI* and *TI* were estimated according to Equation (2) [7] and (3) [18]:

(2) $$\;AI=\frac{\left[\left(C12:0\right)+4\times \left(C14:0\right)+\;\left(C16:0\right)\right]}{\sum MUFA+\sum \omega -3\;+\sum \omega -6}$$

(3) $$\;TI=\frac{C14:0+C16:0+C18:0}{0.5\times (\sum MUFA)+3\times \sum \omega -3+0.5\times \sum \omega -6+\left(\frac{\sum \omega -3}{\omega -6}\right)}$$

where myristic acid (*C*14:0), palmitic acid (*C*16:0), stearic acid (*C*18:0), oleic acid (*C*18:1), linoleic acid (*C*18:2), and α-linolenic acid (*C*18:3). *MUFA* (monounsaturated fatty acids sum); while Σ*ω*−3 is the sum of the polyunsaturated fatty *ω*−3 acids, and Σ*ω*−6 includes the sum of the polyunsaturated fatty *ω*−6 acids.

On the other hand, *H*/*H*, the ratio between hypocholesterolemic and hypercholesterolemic fatty acids, was calculated according to Equation (4) [18], while *COX* value was based on Equation (5) [7] as follows:

(4) $$H/H=\frac{C18:1+C18:2+C18:3}{C14:0+C16:0}$$

(5) $$COX\;value=\frac{\left[C18:1\;(\%)+10.3\times C18:2\;(\%)+21.6\times C18:3\;(\%)\right]}{100}$$

Additionally, in this work, SFA: saturated fatty acids (*C*16:0 + *C*18:0); USFA: unsaturated fatty acids (*C*18:1n9c + *C*20:1); and PUFA: polyunsaturated fatty acids (*C*18:2n6c) were considered.

#### 3.3.4. Quantification of α-Tocopherol Content

The methodology described by Miranda et al. [52] was used to determine the tocopherol content. Pisco lipophilic fractions (25.0 mg) were mixed for 1 h with butylated hydroxytoluene in methanol (100 μL BHT concentration: 1 mg/mL). The filtered supernatant (0.45 μm) was injected for analysis. To carry out the analysis of α-tocopherol, HPLC-fluorescence was used with a Kromasil C18 column (250  ×  4.6 mm, 5 μm), and acetonitrile: methanol (1:1) as mobile phase, with a flow of 1.2 mL/min. Levels were reported as μg of α-tocopherol/g of dried pisco grape pomace, performing analysis in triplicate.

#### 3.3.5. Total Polyphenol Content (TPC)

According to the methodology of Bail et al. [58], the total polyphenol content in the pisco lipophilic fractions was established. First, to extract the polyphenol content, 200 mg of lipophilic fractions was used with 1.5 mL of 90% methanol, followed by agitation for 5 min and centrifugation at 5000 rpm for 5 min. This procedure was performed three times, and all methanolic extracts were combined and evaporated until dry. Next, the resultant sample was reconstituted to 250 μL with 10% methanol. The analysis was performed using a multiplate reader (Perkin-Elmer, Victor TM χ3, Turku, Finland). Using 96-well microplates, the reaction was conducted by mixing 15 μL of extract, 100 μL of Folin–Ciocalteau (0.2 N) reagent, and 100 μL sodium carbonate (60 g/L), and allowed to stand for 90 min. After that, the absorption was measured at 750 nm against a blank sample (15 μL of solvent). Gallic acid was used as a standard for the calibration curve, expressing TPC results as mg of gallic acid equivalent (GAE)/g of dried pisco grape pomace, executing all measurements in triplicate.

### 3.4. Antioxidant Potential of Lipophilic Fractions from Pisco Grape Pomaces

#### 3.4.1. DPPH Assay

Methodologies reported by Espín et al. [59] and Grajeda-Iglesias et al. [55], with some modifications, were used to determine the antioxidant potential by DPPH assay. Extractions using 5, 25, 50, and 100 mg were performed with 200 μL of methanol and mixed in an orbital shaker (OS-20, Boeco, Boelter Electronic GmbH, Hamburg, Germany) for 1 h with a centrifugation step for 5 min at 5000 rpm. The supernatant was measured directly, while the remaining oil was extracted for 1 h with ethyl acetate. Solutions of 120 μM DPPH in methanol and ethyl acetate were prepared. 25 μL of the sample was added to 180 μL of each DPPH solution in transparent 96-well plates, allowing them to react for 30 min in a multiplate reader (Perkin-Elmer, Victor TM χ3, Turku, Finland). The absorbance was read at 517 nm, and the results were represented as the scavenging activity (%), according to Equation (6) for each solvent:

(6) $$Scavening\;activity\left(\%\right)=\frac{Abs\;Control-Abs\;Sample}{Abs\;Control}\times 100$$

where *Abs Control* was the absorbance resulting from the reaction using ethyl acetate or methanol in the presence of the DPPH radical, while *Abs Sample* was the absorbance when each concentration of the fraction was evaluated in the reaction.

#### 3.4.2. ORAC Assay

Zhang et al. [60] methodology developed for the ORAC assay was used with modifications. The mixing of 50 mg of each lipophilic fraction with different solvents (acetone, ethyl acetate or *n*-hexane) was carried out for 1 h and centrifuged for 5 min/5000 rpm. After using 7% MCD (Methyl-beta-cyclodextrin, Sigma Aldrich), the samples were diluted and mixed for 1 h in an orbital shaker (OS-20, Boeco, Germany). Next, using 96-well black polystyrene microplates, 25 μL of the sample was mixed with 200 μL of fluorescein (100 μM in phosphate buffer, 75 mM, pH 7.4), and incubated for 20 min at 37 °C. Then, 25 μL of AAPH 0.36 M (2,2′-Azobis dihydrochloride of 2-methylpropionamide) was added to start the reaction. Fluorescein was read every 60 s at 510 nm in a multiplate reader (PerkinElmer, Victor TM χ3, Turku, Finland), until the fluorescein readings declined to less than 5% of the initial value. Results were expressed as μmol of Trolox equivalents (TE)/mg of extract, quantified by the difference in the area under the curve between the sample and the blank of the fluorescein decay curve and the Trolox calibration curve. All measurements were executed in triplicate.

### 3.5. Statistical Analysis

The experiments were conducted in triplicate, and the data were represented as the mean values ± standard deviation (SD). Results were evaluated through statistical analysis using Statgraphics Plus^®^ software for Windows version 5.1 (Statistical Graphics Corp., Herndon, VA, USA). Differences between extraction methods and harvests were assessed using the least significant difference (LSD) test with a significance level of α = 0.05 and a confidence interval of 95%.

## 4. Conclusions

The extraction method and conditions affected the oil yield, fatty acid profile, total polyphenol, vitamin E content, and antioxidant potential. Quality indicators, such as the refractive index, did not exhibit statistically significant differences between extraction techniques. At the same time, PV did show that the high temperatures of Randall could promote effects on the lipid biocompound stability, favoring their oxidation. The fatty acid profile depicted a small ratio of saturated FA/unsaturated FA. It was a helpful indicator for studying the functional potential by its correlation with the atherogenicity, thrombogenicity indices, and the H/H ratio. PLE showed a decrease in α-tocopherol content, but exhibited the highest ORAC values, showing better performance and preservation of antioxidant compounds. Global results could provide us with guidelines to envision the applications of pisco grape pomace lipophilic fractions in different areas, considering the effect of different extraction methods on chemical composition. 

## Figures and Tables

**Table 1:** Yields for lipophilic fractions from pisco grape pomace.

Harvest	Randall (%)	PLE (%)
2019	9.12 ± 1.47 ^aA^	15.20 ± 1.33 ^bB^
2021	9.58 ± 0.85 ^aA^	13.42 ± 0.90 ^bAB^
2023	9.03 ± 1.49 ^aA^	11.79 ± 1.16 ^aA^

Note: Unit: g extract /100 g of grape pomace. Values are expressed as means ± SD of triplicate. Values with different lowercase letters in the same column indicate a comparison between extraction techniques in each harvest (*ρ* < 0.05). Values with different uppercase letters indicate a comparison among harvests (*ρ* < 0.05). PLE: Pressurized Liquid Extraction.

**Table 2:** Quality parameters for lipophilic fractions of pisco grape pomace by different extraction techniques and harvests.

Extraction Method	Randall		PLE
Refractive index *	2019	1.4561 ± 0.0054 ^a^	1.4560 ± 0.0023 ^a^
	2021	1.4484 ± 0.0240 ^a^	1.4634 ± 0.0012 ^a^
	2023	1.4620 ± 0.0036 ^a^	1.4569 ± 0.0035 ^a^
	2019	21.70 ± 2.94 ^a^	14.51 ± 0.29 ^b^
Peroxide value (PV)	2021	27.24 ± 4.18 ^a^	22.97 ± 1.96 ^b^
	2023	28.95 ± 1.43 ^a^	21.66 ± 0.86 ^b^
Water activity *(a_w_)	2019	0.3741 ± 0.010 ^a^	0.4229 ± 0.018 ^b^
	2021	0.4357 ± 0.053 ^a^	0.4278 ± 0.053 ^a^
	2023	0.4034 ± 0.005 ^a^	0.3966 ± 0.004 ^a^

Note: Values are expressed as means ± SD of triplicate. *: dimensionless; PV: meq O_2_/kg oil. Values with different lowercase letters indicate comparison between extraction techniques for each harvest regarding quality parameter (*ρ* < 0.05). PLE: Pressurized Liquid Extraction.

**Table 3:** Relative content (%) of fatty acids in pisco grape pomace lipophilic fractions by different extraction techniques and harvests.

Fatty Acid/ Extraction Method and Harvest	Palmitic (C16:0)	Stearic (C18:0)	Oleic (C18:1n9c)	Linoleic (C18:2n6c)	Eicosenoic (C20:1)	Saturated Fatty Acids (S)	Monounsaturated Fatty Acids	Polyunsaturated Fatty Acids	Unsaturated Fatty Acids (U)	Ratio S/U
PLE-2019	8.64 ± 0.05 ^d^	3.81 ± 0.02 ^c^	20.90 ± 0.06 ^c^	65.63 ± 0.12 ^a^	1.02 ± 0.12 ^a^	12.45 ± 0.06 ^a^	21.92 ± 0.06 ^a^	65.63 ± 0.12 ^a^	87.55 ± 0.06 ^a^	0.14 ± 0.00 ^a^
Randall-2019	8.58 ± 0.09 ^d^	3.79 ± 0.01 ^c^	21.01 ± 0.04 ^c^	65.66 ± 0.18 ^a^	0.95 ± 0.16 ^a^	12.38 ± 0.10 ^a^	21.96 ± 0.12 ^a^	65.66 ± 0.18 ^a^	87.62 ± 0.10 ^a^	0.14 ± 0.00 ^a^
PLE-2021	7.59 ± 0.05 ^ab^	3.58 ± 0.03 ^a^	18.87 ± 0.09 ^b^	69.03 ± 0.15 ^bc^	0.94 ± 0.01 ^a^	11.17 ± 0.07 ^b^	19.85 ± 0.08 ^b^	69.03 ± 0.15 ^a^	88.88 ± 0.15 ^a^	0.13 ± 0.00 ^a^
Randall-2021	7.39 ± 0.04 ^a^	3.64 ± 0.01 ^b^	18.78 ± 0.01 ^b^	69.36 ± 0.07 ^c^	0.84 ± 0.01 ^a^	11.02 ± 0.02 ^a^	19.63 ± 0.01 ^a^	69.36 ± 0.07 ^b^	88.99 ± 0.07 ^a^	0.12 ± 0.00 ^a^
PLE-2023	7.81 ± 0.16 ^b^	3.55 ± 0.03 ^ab^	17.81 ± 0.07 ^a^	69.21 ± 0.21 ^b^	1.62 ± 0.11 ^b^	11.36 ± 0.18 ^a^	19.43 ± 0.05 ^a^	69.21 ± 0.21 ^a^	88.64 ± 0.18 ^a^	0.13 ±0.00 ^a^
Randall-2023	8.15 ± 0.39 ^c^	3.59 ± 0.06 ^a^	18.18 ± 0.55 ^a^	68.62 ± 0.52 ^c^	1.43 ± 0.35 ^b^	11.74 ± 0.45 ^a^	19.60 ± 0.31 ^a^	68.62 ± 0.52 ^a^	88.23 ± 0.40 ^a^	0.13 ± 0.00 ^a^

Note: Values are expressed as means ± SD of triplicate. Values with different lowercase letters in the same column indicate a comparison of all extraction techniques for all harvests (*ρ* < 0.05). PLE: Pressurized Liquid Extraction

**Table 4:** Indicators of functional quality of lipophilic fractions from pisco grape pomace.

Extraction Method and Harvest	AI	TI	H/H	COX Value	USFA/SFA	PUFA/SFA
PLE-2019	0.099 ± 0.000 ^a^	0.285 ± 0.001 ^a^	10.018 ± 0.073 ^a^	6.760 ± 0.012 ^a^	7.031 ± 0.040 ^a^	5.270 ± 0.035 ^a^
Randall-2019	0.098 ± 0.008 ^a^	0.282 ± 0.003 ^a^	10.097 ± 0.124 ^a^	6.763 ± 0.019 ^a^	7.081 ± 0.066 ^a^	5.306 ± 0.056 ^a^
PLE-2021	0.085 ± 0.001 ^b^	0.251 ± 0.002 ^b^	11.580 ± 0.088 ^a^	7.100 ± 0.014 ^a^	7.954 ± 0.056 ^a^	6.166 ± 0.039 ^a^
Randall-2021	0.083 ± 0.000 ^a^	0.248 ± 0.001 ^a^	11.931 ± 0.071 ^b^	7.139 ± 0.006 ^b^	8.071 ± 0.043 ^b^	6.281 ± 0.026 ^b^
PLE-2023	0.092 ± 0.002 ^a^	0.266 ± 0.011 ^a^	10.670 ± 0.619 ^a^	7.089 ± 0.019 ^a^	7.522 ± 0.327 ^a^	6.851 ± 0.264 ^a^
Randall-2023	0.088 ± 0.002 ^a^	0.256 ± 0.004 ^a^	11.149 ± 0.272 ^a^	7.131 ± 0.021 ^a^	7.805 ± 0.136 ^a^	6.094 ± 0.113 ^a^

Note: SFA: saturated fatty acids; USFA: unsaturated fatty acids; PUFA: polyunsaturated fatty acids; TI: thrombogenic index = [C16:0 + C18:0]/[(0.5 (C18:1n9 + C20:1)) + (0.5⋅(C18:2n6))]. AI: atherogenic index = [ C16:0]/[(C18:1n9 + C20:1) + (C18:2n6)]. Hypocholesterolemic FA (H)/hypercholesterolemic FA (H) = (C18:2n6)/(C16:0). COX value = 10.3⋅C18:2n6/100, refers to oxidizability, based on unsaturated fatty acid percentages in the oils. PLE: Pressurized Liquid Extraction. Values with different lowercase letters indicate comparison between extraction techniques for each harvest regarding quality parameter (*ρ* < 0.05).

**Table 5:** α-tocopherol content in lipophilic fractions by Randall and PLE methods.

Harvest	Randall α-Tocopherol (μg/g)	PLE α-Tocopherol (μg/g)
2019	180.53 ± 6.49 ^a^	86.63 ± 15.97 ^b^
2021	644.68 ± 12.49 ^b^	156.02 ± 1.39 ^a^
2023	330.88 ± 3.18 ^b^	172.92 ± 11.45 ^a^

Note: Values are expressed as means ± SD of triplicate. Different letters as superscripts indicate comparison to each extraction technique in each harvest (*ρ* < 0.05). PLE: Pressurized Liquid Extraction.

**Table 6:** Total polyphenol content in pisco grape pomace lipophilic fractions by different extraction techniques and harvests.

Harvest	Randall	PLE
2019	0.61 ± 0.05 ^a^	0.85 ± 0.05 ^b^
2021	0.62 ± 0.23 ^a^	0.58 ± 0.07 ^a^
2023	0.71 ± 0.07 ^a^	0.80 ± 0.13 ^a^

Note: Values are expressed as means ± SD of triplicate. Different letters as superscripts indicate comparison to each extraction technique in each harvest (*ρ* < 0.05). PLE: Pressurized Liquid Extraction; mg of gallic acid equivalent (GAE)/g of extract.

**Table 7:** Antioxidant potential by ORAC assay of lipophilic fractions by Randall and PLE.

Extraction Method	Harvest	Acetone	Ethyl Acetate	*n*-Hexane
Randall	2019	27.08 ± 2.56 ^a^	48.83 ± 2.03 ^b^	74.61 ± 3.27 ^ab^
	2021	39.70 ± 3.20 ^b^	73.68 ± 1.21 ^c^	75.28 ± 3.93 ^b^
	2023	35.70 ± 0.92 ^b^	36.79 ± 2.58 ^a^	68.56 ± 1.99 ^a^
PLE	2019	68.61 ± 4.67 ^a^	73.59 ± 4.92 ^a^	89.37 ± 2.08 ^a^
	2021	119.10 ± 5.38 ^b^	120.92 ± 4.88 ^b^	111.28 ± 4.38 ^b^
	2023	65.89 ± 3.41 ^a^	80.58 ± 1.91 ^a^	119.55 ± 2.87 ^c^

Note: Values are expressed as means ± SD of triplicate. Different letters as superscripts indicate a comparison for each solvent in each harvest (*p* < 0.05). PLE: Pressurized Liquid Extraction; µmol of trolox equivalent (TE)/g of fraction.

## Data Availability

The data used or analyzed during the current study are available from the corresponding author upon reasonable request.
